# CCAAT/Enhancer Binding Protein-delta (C/EBP-delta) regulates cell growth, migration and differentiation

**DOI:** 10.1186/1475-2867-10-48

**Published:** 2010-12-09

**Authors:** Xueyan Yu, Junling Si, Yingjie Zhang, James W DeWille

**Affiliations:** 1 Department of Veterinary Biosciences, Ohio State University College of Veterinary Medicine and OSU Comprehensive Cancer Center, 1925 Coffey Road, Columbus Ohio, 43210, USA

## Abstract

**Background:**

CCAAT/enhancer binding protein-delta (C/EBP-delta) is a member of the highly conserved C/EBP family of basic region leucine zipper transcription factors. C/EBP family members regulate cell growth and differentiation and "loss of function" alterations in C/EBPs have been reported in a variety of human cancers. C/EBP-delta gene expression is upregulated by G_0 _growth arrest, IL-6 family cytokines and endotoxin treatments. C/EBP-delta exhibits properties of a tumor suppressor gene, including reduced expression and promoter methylation-induced silencing in transformed cell lines and primary tumors. In addition, C/EBP-delta gene expression is repressed by c-Myc, an oncogene that is over-expressed in a wide range of human cancers. "ChIP-chip" studies demonstrated that C/EBP-delta functions as a transcriptional activator of target genes that function in intracellular signal transduction, transcription, DNA binding/repair, cell cycle control, cell adhesion, and apoptosis. Despite progress in determining the biochemical functions of C/EBP-delta, the specific cellular defects that are induced by C/EBP-delta "loss of function" alterations are poorly understood. This study investigated the impact of C/EBP-delta "loss of function" alterations on growth arrest, migration/invasion and differentiation in nontransformed mouse mammary epithelial cells (MECs) and primary mouse embryo fibroblasts (MEFs).

**Results:**

C/EBP-delta siRNA transfected MECs exhibited ~90% reduction in C/EBP-delta mRNA and protein levels. C/EBP-delta siRNA treatment resulted in defective growth arrest as demonstrated by persistently elevated BrdU labeling, ^3^H-thymidine incorporation and cyclin D1 levels in response to growth arrest treatments. C/EBP-delta siRNA treatment also resulted in increased migration/invasion and defective differentiation. C/EBP-delta knockout MEFs exhibited defective growth arrest and increased proliferation/migration. Re-introduction of C/EBP-delta expression restored the growth arrest response of C/EBP-delta knockout MEFs. Finally, deletion of the C/EBP-delta DNA binding domain or the C/EBP-delta bZIP domain resulted in the loss of C/EBP-delta growth inhibition in clonogenic assays.

**Conclusions:**

This study demonstrates that C/EBP-delta functions in the regulation of critical cell fate determining programs such as growth arrest, migration, and differentiation. These results support the tumor suppressor function of C/EBP-delta and identify potential mechanisms in which "loss of function" alterations in C/EBP-delta could promote cell transformation and tumorigenesis.

## Background

CCAAT/enhancer binding proteins (C/EBPs) are a highly conserved family of basic region leucine zipper (bZip) transcription factors [1]. The C/EBP family includes six family members: C/EBPα, C/EBPβ, C/EBPγ, C/EBPδ, C/EBPε, and C/EBPζ [1,2]. C/EBP proteins exhibit significant amino acid homology (>90%) in the bZip (C-terminal) domain, however, the N-terminal regions of C/EBPs are quite divergent exhibiting <20% sequence homology [1]. C/EBPs influence cell fate by forming homo- or heterodimers with other C/EBP family members as well as other bZip-containing proteins such as Fos, Jun and cyclic AMP response element binding protein (CREB) [1-3]. C/EBPs also interact with cell cycle regulators such as Retinoblastoma protein (Rb), E2F, cyclin dependent kinase4 (CDK4) and p21 via the C/EBP N terminal region [1-3]. C/EBP family members, particularly C/EBPα and C/EBPδ, exhibit cell type specific anti-proliferative activities and, as a result, have been termed "molecular stop signs" [2,4].

C/EBPδ gene expression is highly induced in a wide range of cell types by growth arrest treatments [2]. C/EBPδ is induced in primary human mammary epithelial cells and human and mouse non-transformed mammary epithelial cell lines in response to growth arrest treatments (serum and growth factor withdrawal, contact inhibition) and IL-6 family cytokine treatment [5-8]. C/EBPδ is also induced in human prostate cancer derived cells (LnCAP) and in KCL22 and K562 chronic myelogenous leukemia (CML) cell lines in response to growth arrest and IL-6 family treatments [9,10]. The induction of C/EBPδ expression in response to growth arrest treatments has functional consequences as ectopic C/EBPδ expression induces growth arrest in mammary epithelial, prostate, CML and AML derived cell lines [6,9-11]. In addition, C/EBPδ knockout female mice exhibit increased mammary epithelial cell proliferation and ductal hyperplasia, demonstrating that reduced C/EBPδ expression results in mammary epithelial cell growth abnormalities *in vivo *[12].

C/EBPδ "loss of function" alterations have been demonstrated in a number of human cancers. We reported that C/EBPδ expression is reduced in 32% (18/57) of primary human breast tumors, a finding consistent with Serial Analysis of gene Expression (SAGE) results from Polyak and coworkers [13-15]. The association between reduced C/EBPδ expression and mammary tumorigenesis has also been demonstrated in mammary tumor prone MMTV/c-neu transgenic mice and rodent carcinogen-induced mammary tumors, indicating that reduced C/EBPδ expression is relatively common in mammary epithelial cell tumors regardless of species or transforming event [16,17]. Mechanistic studies indicate that hypermethylation and site-specific methylation within the C/EBPδ proximal promoter is associated with reduced C/EBPδ gene expression in the human SUM-52PE breast cancer cell line and in primary human breast tumors [13]. Silencing of gene expression by epigenetic promoter hypermethylation has been previously reported for a number of tumor suppressor genes including Rb, p16, p21, BRCA1 and VHL [18]. These results demonstrate that C/EBPδ shares cancer related mechanisms of gene silencing with established tumor suppressor genes.

C/EBPs function as transcriptional regulators in the differentiation of a variety of cell types, including adipocytes, mammary epithelial cells, myeloid cells, keratinocytes and hepatocytes [1,19]. Sequential expression of C/EBP δ, β and α has been extensively documented in adipocyte differentiation [20]. C/EBPα, C/EBPβ knockout mice and C/EBPβ/δ double knockout mice exhibit reduced *in vitro *adipocyte differentiation and reduced *in vivo *lipid accumulation in adipose tissue [21,22]. C/EBPδ is also important in differentiation of cells in the myeloid lineage as ectopic C/EBPδ expression induces growth arrest and differentiation in chronic myelogenous leukemia cell lines [10]. In contrast, inhibiting C/EBPδ expression suppresses cytokine-induced granulocytic differentiation of human myeloid leukemia cells [23].

The overall goal of this study was to investigate the influence of C/EBPδ on growth arrest, migration, invasion and differentiation. Alterations in these fundamental cell programs are common in cancer cells [24]. The results demonstrated that reducing C/EBPδ expression impairs mammary epithelial cell (MEC) growth arrest, increases MEC migration/invasion and reduces MEC differentiation. In addition, C/EBPδ knockout MEFs also exhibited defective growth arrest and increased migration compared to C/EBPδ wild type MEFs. These results provide mechanistic insights into the role of C/EBPδ "loss of function" alterations" in cell transformation and tumorigenesis.

## Results

### Reducing C/EBPδ expression by C/EBPδ siRNA treatment results in defective HC11 mammary epithelial cell growth arrest

**Figure 1:**
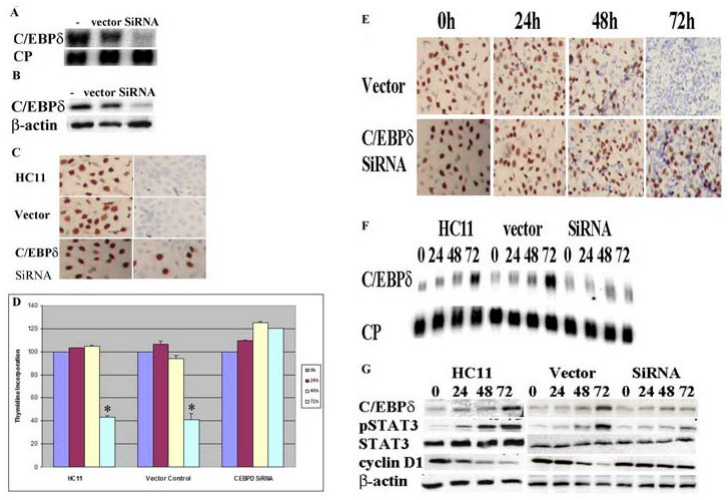
**Reducing C/EBPδ expression by C/EBPδ siRNA treatment results in defective HC11 mammary epithelial cell growth arrest**. (A) C/EBPδ mRNA levels were assessed in parental (nontransfected) HC11 cells ("-"), empty vector (*pSilencer *™ 2.1) transfected HC11 controls ("vector") and C/EBPδ siRNA transfected ("siRNA") HC11 cells growth arrested by serum and growth factor withdrawal. Northern blots were probed with ^32^P-labelled C/EBPδ and cyclophilin (CP, loading control) cDNA probes. (B) C/EBPδ protein levels were assessed in parental (nontransfected) HC11 cells (-), empty vector transfected HC11 controls (vector) and C/EBPδ siRNA transfected (siRNA) HC11 cells growth arrested by serum and growth factor withdrawal. Western blots were probed with primary antibodies against C/EBPδ and β-actin (loading control). (C) Serum and growth factor withdrawal induced growth arrest assessed by BrdU labeling (brown). HC11 cells were cultured under exponentially growing (first panel) and 48 hr growth arrested (second panel) conditions. Cells were counterstained with hematoxylin (blue). (D) Contact inhibition (growth arrest) assessed by [^3^H]-thymidine incorporation. HC11 nontransfected control, HC11 vector control and HC11 C/EBPδ siRNA transfected cells were grown to ~80% confluence (0 hr) in complete growth media (CGM) and then maintained in CGM. [^3^H]-thymidine incorporation (cpm) was assessed at 0, 24 48 and 72 hours. (E) Contact inhibition (growth arrest) assessed by BrdU incorporation (brown). Cells were treated as described in "D" above. BrdU incorporation was assessed at the designated time points. (F) Contact inhibition (growth arrest) induction of C/EBPδ mRNA levels. Total RNA was isolated at the designated time points and Northern blots were probed with ^32^P-labelled C/EBPδ and cyclophilin (CP, loading control) cDNA probes. (G) Whole cell proteins were isolated at designated time points and Western blots were probed with primary antibodies against STAT3, pSTAT3, C/EBPδ, cyclin D1 and β-actin (loading control). * = significant different from C/EBPδ siRNA treated group at 72 hours at p < 0.05.

Previous reports from our lab and others have demonstrated that C/EBPδ gene transcription is highly induced during G_0 _growth arrest and C/EBPδ gene expression is reduced in breast cancer cell lines and primary breast tumors [7,8,13,15]. To investigate the functional role of C/EBPδ in mammary epithelial cell G_0 _growth arrest we developed stably transfected C/EBPδ siRNA expressing and vector control HC11 cell lines. Stable C/EBPδ siRNA expressing HC11 cells exhibited ~90% reduction in C/EBPδ mRNA and protein levels compared to HC11 parental and vector controls (Figure 1AB). The influence of C/EBPδ expression HC11 cell growth was investigated by culturing HC11 cell lines under exponentially growing or growth arrest conditions. Cell growth (cell proliferation) status was assessed using bromodeoxyuridine (BrdU) *in situ *labeling as an indicator of S phase DNA synthesis. The number of BrdU positive nuclei detected in all 3 HC11 cell lines cultured under exponentially growing cell culture conditions was approximately similar, suggesting that C/EBPδ does not play a major role under conditions in which cells are actively proliferating (Figure 1C, left column). The number of BrdU positive nuclei was dramatically reduced, however, when parental and vector control HC11 cells were cultured under growth arrest conditions for 48 hours (Figure 1C, right panel). In contrast, the number of BrdU positive nuclei was sustained at relatively elevated levels in the C/EBPδ siRNA expressing HC11 cells after cultured under growth arrest conditions for 48 hours (Figure. 1C, right panel). These results indicate that serum and growth factor withdrawal activates the growth arrest response in C/EBPδ expressing HC11 cells, but HC11 cells with reduced C/EBPδ expression retain the capacity to proliferate in serum and growth factor deficient media.

We next investigated the effect of reduced C/EBPδ expression on contact mediated growth arrest. To assess contact mediated growth arrest near (~80%) confluent HC11 cell lines were cultured in media containing serum and growth factors (complete growth media, CGM) for up to 72 hours. [^3^H] thymidine incorporation and BrdU labeling were assessed as indicators of proliferation post confluence. HC11 parental and vector control cells exhibited a significant decline in [^3^H] thymidine incorporation after 72 hours (Figure 1D). In contrast, [^3^H] thymidine incorporation remained elevated after 72 hours post confluence in C/EBPδ siRNA transfected HC11 cells (Figure 1D). Consistent with the [^3^H] thymidine incorporation data, the BrdU *in situ *labeling results also demonstrated that C/EBPδ siRNA transfected HC11 cells exhibit impaired contact inhibition (increased BrdU positive nuclei) at 72 hours compared to vector transfected controls (Figure 1E). These results demonstrate that contact mediated growth arrest is defective in C/EBPδ siRNA transfected HC11 cells.

We next assessed the association between contact mediated growth arrest and cellular markers of proliferation and growth arrest. Consistent with previous reports from our laboratory, C/EBPδ mRNA levels increase as parental and vector control HC11 cells progress from sub confluence (0 hr) to full confluence (72 hrs) (Figure 1F) [5,25]. In contrast, C/EBPδ mRNA levels are not significantly induced in C/EBPδ siRNA expressing HC11 cells cultured under the same conditions of confluence (Figure 1F). Previous reports from our laboratory and others have demonstrated that activated (phosphorylated) STAT3 (pSTAT3) is the principal transcriptional activator of C/EBPδ gene expression in mouse and human cells in response to growth arrest conditions [25-28]. Reports from our lab and others have also demonstrated that pSTAT3 levels increase in growth arrested, nontransformed cells, but alterations in pSTAT3 levels and pSTAT3 mediated C/EBPδ gene expression are demonstrable in cancer cell lines [8,9,13,25,29-32]. Western blot analysis demonstrated that as parental and vector control HC11 cell lines approach contact mediated growth inhibition (48-72 hours), pSTAT3 and C/EBPδ protein levels increase and the levels of Cyclin D1, a marker of cell cycle proliferation, decline (Figure 1G). In contrast, pSTAT3 and C/EBPδ protein levels remain relatively low and Cyclin D1 levels remain elevated in C/EBPδ siRNA treated HC11 cells (Figure 1G). Taken together, these results demonstrate that reducing C/EBPδ expression by siRNA treatment results in defective growth arrest in nontransformed HC11 mammary epithelial cells.

### Reducing C/EBPδ expression increases HC11 cell migration and invasion

**Figure 2:**
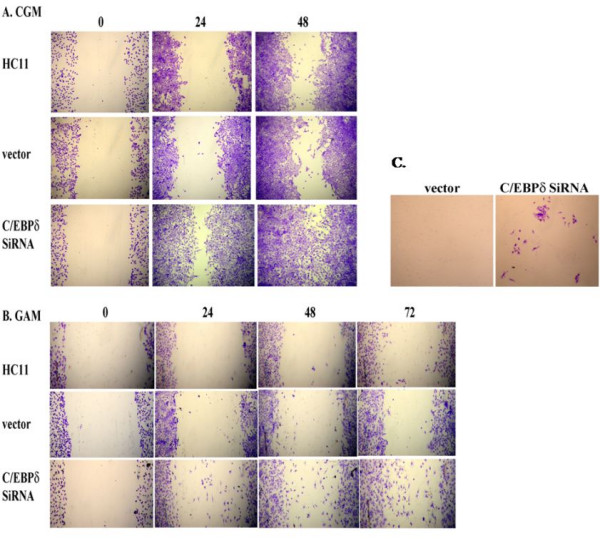
**Reducing C/EBPδ expression increases HC11 cell migration and invasion**. (A) HC11 nontransfected cells, HC11 stably transfected vector (*pSilencer *™ 2.1 neo) control ("vector") cells and HC11 C/EBPδ siRNA treated cells were grown to confluence in CGM. A 200 μl pipet tip was used to produce an open area or "scratch" in the confluent monolayers and migration into the open area assessed at 0 h, 24 h and 48 h by crystal violet staining. (B) HC11 cell lines were grown to confluence in CGM and then switched to growth arrest media (GAM, 0.1% FBS) for 24 hours prior to the creation of the open area in the cell monolayers. Migration into the open area in the GAM cultured cells was assessed as described in "A" above. (C) HC11 vector control and C/EBPδ siRNA treated cells (1 × 10^6^) were suspended in serum-free media and cultured on the inner (top) chamber of the insert; serum containing media was placed in the outer (lower) chamber of the insert (Chemicon Cell Invasion Assay Kit). Plates were incubated for up to 6 days at 37°C. Migration of cells to the lower surface of the membrane was assessed by staining with crystal violet and photographed. Results presented are representative of 3 independent experiments with duplicates.

We previously demonstrated by "ChIP-chip" assay that C/EBPδ activates target genes that function cell adhesion, including integrinβ8 (ITGB8), protocadherin 9 (PCDH9) and glycoprotein V (GP5) [33]. We hypothesized that in addition to defective growth control, "loss of function" alteration in C/EBPδ may also influence cell migration. HC11 cell migration was assessed using the *in vitro *"scratch" assay, an assay that assesses the capacity of cells to migrate into an open area created by "scratching" a confluent cell monolayer [34-36]. The "scratch' assay was performed on confluent monolayers of HC11 cells cultured in complete growth media and migration into the open area assessed at 0, 24 and 48 hours post-scratch. The results demonstrated that C/EBPδ siRNA transfected HC11 cells exhibited enhanced migration into the open area of the cell monolayer compared to parental or vector control cells (Figure 2A). To determine the influence of exogenous growth factors on cell migration the scratch assay was performed with cells cultured at confluence in media lacking serum and growth factors (Growth arrest media, GAM). Although overall migration was significantly reduced in all cells cultured in GAM, the number of cells migrating into the open area was increased in the C/EBPδ siRNA transfected HC11 cells compared to the parental and vector control groups (Figure 2B). These results demonstrate that reducing C/EBPδ expression is associated with increased cell migration, a property of transformed cells that is linked to metastasis [35].

To determine if the increased cell migration capability exhibited by C/EBPδ siRNA treated HC11 cells also increased cell invasion we performed cell invasion assays. C/EBPδ siRNA expressing and vector control HC11 cells were cultured in GAM on extracellular matrix (ECM) coated inserts and invasion was assessed by crystal violet staining of cells traversing to the reverse side of the insert. Although nontransformed HC11 cell line is minimally invasive in this assay, the results demonstrated that siRNA mediated reduction of C/EBP expression increased HC11 cell invasion through the ECM, indicating that "loss of function" alterations in C/EBPδ are associated with enhanced cell invasion (Figure 2C).

### Reducing C/EBPδ expression inhibits HC11 mammary epithelial cell differentiation

**Figure 3:**
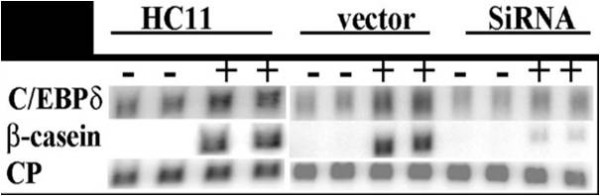
**Reducing C/EBPδ expression inhibits HC11 mammary epithelial cell differentiation**. HC11 nontransfected control cells, HC11 stably transfected vector (*pSilencer*™ 2.1 neo) control ("vector") cells and HC11 C/EBPδ siRNA treated cells were grown to confluence in CGM. Cells were cultured in CGM without lactogenic hormones (-) or in CGM plus lactogenic hormones (+) for 4 days. Total cellular RNA was isolated and Northern blot analysis performed. Northern blots were probed with ^32^P-labelled C/EBPδ and β-casein expression cDNA probes. CP was used as the loading control. Results presented are for mRNA levels from duplicate samples analyzed in this experiment. The results presented are representative of 3 experiments.

Early reports associated C/EBPδ expression with adipocyte differentiation and subsequent studies established that C/EBPδ plays a key regulatory role in adipocyte and granulocyte differentiation [10,37]. Since C/EBPδ is involved in the differentiation of cell types of various lineages we hypothesized that loss of C/EBPδ expression would alter mammary epithelial cell differentiation. Confluent HC11 cells were treated with a media containing lactogenic hormones and the influence of C/EBPδ expression on HC11 cell differentiation was assessed by Northern blot analysis of β-casein mRNA, a marker of mammary epithelial cell differentiation [17]. The results demonstrated that β-casein mRNA levels increased in the HC11 parental and vector control cell lines in response to lactogenic hormone treatment ("+" columns) compared to control cell lines cultured in the absence of lactogenic hormones ("-" columns)(Figure 3). In contrast, lactogenic hormone induction of β-casein mRNA levels in C/EBPδ siRNA transfected HC11 cells was minimal, ~20% of that observed in the C/EBPδ expressing control cell lines (Figure 3). These results demonstrate that reducing endogenous C/EBPδ levels is associated with defective HC11 mammary epithelial cell differentiation.

### C/EBPδ knockout (C/EBP -/-) mouse embryo fibroblasts (MEFs) exhibit defective growth arrest and increased migration

**Figure 4:**
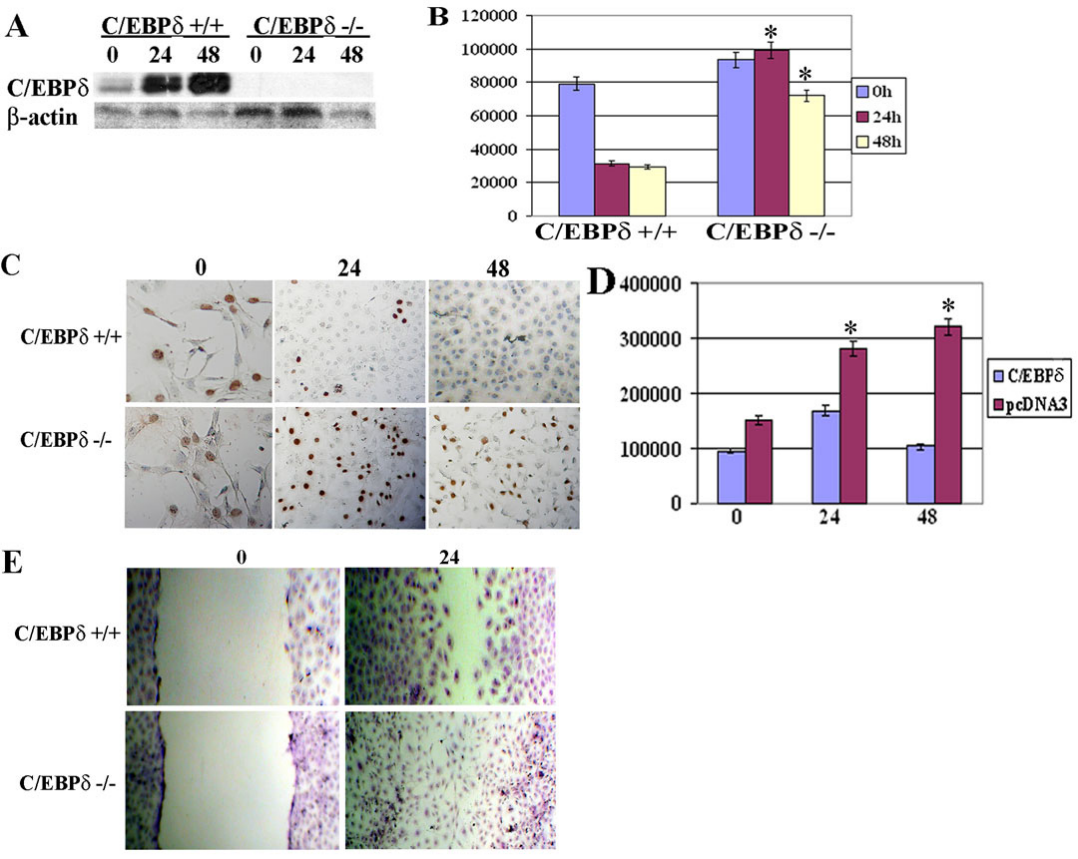
**C/EBPδ knockout (C/EBP -/-) mouse embryo fibroblasts (MEFs) exhibit defective growth arrest and increased migration**. (A) C/EBPδ protein levels in C/EBPδ +/+ and C/EBPδ -/- MEFs. C/EBPδ +/+ and C/EBPδ -/- MEFs were cultured in MCGM to ~80% confluence and then switched to GAM. Whole cell lysates were isolated and western blots performed at 0 (near confluence), 24 and 48 hrs. Western blots were probed with anti- C/EBPδ and β-actin antibodies. (B) [^3^H]-thymidine incorporation. C/EBPδ +/+ and C/EBPδ -/- MEFs were treated as described in "A" above. [^3^H]-thymidine incorporation assessed at 0, 24 and 48 hrs. * = significantly different from C/EBPδ +/+ treatment group at 24 and 48 hours at p < 0.05. (C) BrdU labeling. C/EBPδ +/+ and C/EBPδ -/- MEFs were treated as described in "A" and BrdU labeling detected by in situ immunocytochemisty. (D) C/EBPδ Rescue. C/EBPδ -/- MEFs were transfected with pcDNA3 (empty vector control) or with full length C/EBPδ cloned into pcDNA3. Confluent cells were cultured in MCGM and [^3^H]-thymidine incorporation assessed at 0, 24 and 48 hrs post-confluence. The values presented are mean ± S.E.M from three independent experiments. * = significantly different from MEFs transfected with C/EBPδ expression vector at 24 and 48 hours at p < 0.05. Migration assay. C/EBPδ +/+ and C/EBPδ -/- MEF confluent monolayers cultured in MCGM were "scratched" with a 200 μL pipet tip as described in the Methods section. Migration of cells into the open area was assessed at 0 and 24 hrs by crystal violet staining.

Primary mouse embryo fibroblasts (MEFs) have been extensively used as a model system to investigate the role of specific gene deletions in cell biology [38]. We hypothesized that C/EBPδ -/- MEFs could serve as a useful model to investigate the tumor suppressor functions of C/EBPδ. C/EBP +/+ and C/EBP -/- MEFs were grown to near confluence (0 hr), switched from MCGM to GAM, and growth arrest induction of C/EBPδ protein levels assessed. C/EBPδ +/+ MEFs exhibited marked induction of C/EBPδ protein levels 24 and 48 hours after exposure to GAM (Figure 4A). In contrast, C/EBPδ protein levels were undetectable in C/EBPδ -/- MEFs cultured in GAM, confirming the total abrogation of C/EBPδ expression in C/EBPδ -/- MEFs (Figure 4A). Next, we cultured near confluent (~80% confluent) C/EBPδ +/+ and C/EBPδ -/- MEFs in media deficient in serum and growth factors (growth arrest media, GAM) and assessed the growth arrest response using [^3^H] thymidine incorporation and BrdU labeling as S phase (proliferation) indicators. The results demonstrated that C/EBPδ +/+ MEFs exhibited a rapid, time dependent (within 24 hrs) decline in [^3^H] thymidine incorporation following exposure to GAM (Figure 4B). This decline in [^3^H] thymidine incorporation persisted in the C/EBPδ +/+ MEFs at the 48 hr time point (Figure 4B). In contrast, [^3^H] thymidine incorporation remained elevated in the C/EBPδ -/- MEFs after prolonged (48 hours) of culture in GAM (Figure 4B). Similarly, BrdU labeling declined in C/EBPδ +/+ MEFs after 24 and 48 hours of culture in GAM (Figure 4C). BrdU labeling remained elevated in C/EBPδ -/- MEFs after 24 and 48 hours of culture in GAM (Figure 4C). To further investigate the role of C/EBPδ in contact-mediated growth arrest C/EBPδ -/- MEFs were transfected with an empty expression plasmid (pcDNA3) or a C/EBPδ expression plasmid and cultured in MEF Complete Growth Media (MCGM). C/EBPδ -/- MEFs receiving the C/EBPδ expression plasmid exhibited decreased [^3^H] thymidine incorporation, consistent with growth inhibition (Figure 4D). In contrast, [^3^H] thymidine incorporation continued to increase in C/EBPδ -/- MEFs transfected with the empty pcDNA3 control vector (Figure 4D). These results demonstrate that C/EBPδ plays a key role in activating growth arrest in MEFs. We next investigated the influence of C/EBPδ on MEF migration using the *in vitro *scratch assay. The results demonstrated that a uniform open area was created on the C/EBPδ -/- and C/EBPδ -/- MEF cell monolayers at t = 0 (Figure 4E). After 24 hours, however, the C/EBPδ -/- MEFs demonstrated a markedly elevated migration into the open area compared to C/EBPδ +/+ MEFs (Figure 4E). These results are consistent with the results presented in Figure 2 and further demonstrate that "loss of function" alterations in C/EBPδ enhance cell migration.

### C/EBPδ DNA binding and bZIP domains function in the suppression of HC11 cell colony growth

**Figure 5:**
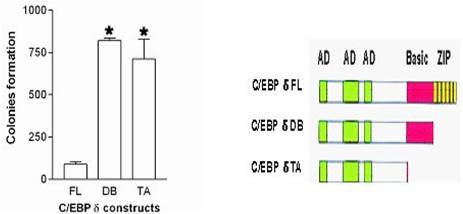
**C/EBPδ transcriptional activator function is required for suppression of HC11 mammary epithelial cell colony growth**. HC11 cells were transfected with expression constructs encoding C/EBPδ cDNA full length (FL), C/EBPδ bZIP domain deleted (DB) or C/EBPδ bZIP + DNA binding domain deleted (TA) constructs. HC11 cells transfected with the pcDNA3 vector alone yielded ~1,000 colonies (positive control). For all clonogenic assays, cells were cultured for 15 days in Complete Growth Media (CGM) plus G418. Surviving colonies were stained with a 0.5% crystal violet/20% methanol solution and counted. * = significantly different from HC11 cells transfected with the full length (FL) C/EBPδ expression vector at p < 0.05.

C/EBPs are organized in a domain structure composed of an N terminal transactivation domain, regulatory domain and highly conserved C-terminal basic region (BR) and leucine zipper (bZIP) domains [2]. CEBPs function primarily as DNA binding transcriptional activators [1,2]. To determine if the C/EBPδ transcriptional activation function plays a role in colony growth HC11 cells were transfected with C/EBPδ full length and C/EBPδ bZIP and C/EBPδ BR + bZIP deletion constructs and colony growth (clonogenic) assays performed. The results demonstrated that the C/EBPδ full length construct suppressed HC11 cell colony growth compared to the transcriptional activation defective C/EBPδ bZIP and C/EBPδ BR + bZIP deletion constructs (Figure 5). These findings demonstrate that the C/EBPδ domains that mediate transcriptional activation (i.e., BR and bZIP) play a major role in C/EBPδ mediated suppression of HC11 cell colony growth.

## Discussion

These findings provide mechanistic insights into the role of C/EBPδ as a tumor suppressor gene and support experimental and clinical reports that have linked "loss of function" alterations in C/EBPδ with aberrant cell growth and tumorigenesis [6,8-11,13-15,39]. Early reports demonstrated that C/EBPδ functioned as a transcriptional activator in growth arrest and differentiation of pre-adipocyte and acute phase responding cells [37,40]. The results from this study extend these results to nontransformed mammary epithelial cells and primary embryo-derived fibroblasts, demonstrating that C/EBPδ regulates cell fate determining programs in a broad range of cell types. Recent studies indicate that C/EBPδ gene expression is induced in hormone responsive breast and prostate cancer cells, but C/EBPδ induction and growth arrest is lost in more advanced, hormone unresponsive breast and prostate cancer cells [39]. In addition to breast and prostate, the association between C/EBPδ, growth control and differentiation extends to myeloid lineage cells. C/EBPδ expression is silenced by promoter hypermethylation in acute myeloid leukemia (AML) patient samples and C/EBPδ is significantly reduced in minimally differentiated, poor prognosis, AML:M0 patient samples [11,41]. Taken together, these results indicate that C/EBPδ "loss of function" alterations are associated with a wide range of "solid" tumors and in leukemia.

In this report we show that reducing C/EBPδ expression by siRNA and gene knockout increased cell migration and invasion, consistent with a role for "loss of function" alterations in C/EBPδ in advanced cancer progression and metastasis. Importantly, these experiments demonstrate that reducing C/EBPδ expression levels increased cell migration under growth arrest conditions, a condition in which C/EBPδ expression is elevated and cell migration is dramatically inhibited (Figure 2B). These findings extend basic studies in which *Slow border cells *(*slbo)*, a Drosophila basic region/leucine zipper (C/EBP) homologue, was shown to function as a regulator of border cell cluster formation, adhesion and migration in the Drosophila ovary [35]. Border cell cluster formation is a complex biological phenomenon that requires activation of adhesion and migration programs. Using "ChIP-chip" assays we previously identified C/EBPδ target genes that function in cell adhesion, including glycoprotein V (GP5), protocadherin 9 (PCDH9) and integrin β8 (ITGB8) [33]. The present results indicate that reducing C/EBPδ expression plays a potentially important role in promoting cell migration, a critical pathogenic event in metastasis.

C/EBPδ domain deletion experiments demonstrated that the transcriptional activator function of C/EBPδ is required for the suppression of HC11 cell colony growth. Colony growth suppression could result from C/EBPδ induced growth arrest or increased apoptosis [6]. We previously identified C/EBPδ target genes with functional roles in cell cycle regulation and apoptosis including septin 7 (SEPT7), regulator of chromosome condensation I (RCCI), DIRAS family GTP-binding Ras-like 3 (DIRAS3) and BCL1-like 1 (BCL2L1) [33]. Transcriptional activation of these C/EBPδ target genes, or indirect activation of C/EBPδ associated gene networks could induce colony growth inhibition [42]. Current studies are investigating the regulation and function of selected C/EBPδ target genes in the control of cell growth, differentiation and migration/invasion.

## Conclusion

An important goal in basic cancer research is to identify biological alterations that promote tumor development and progression and to use this information to design interventions strategies to prevent or treat cancer. The results of this study provide new insights into the function of C/EBPδ in cell biology using a nontransformed mammary epithelial cell line and primary embryo fibroblasts. The results provide new insights into the role of C/EBPδ as a transcriptional activator in growth control, differentiation and migration. These findings suggest that intervention strategies that increase the expression, activation or function of C/EBPδ could have a positive impact or reducing the incidence or impeding the progression of cancer.

## Methods

### Cell culture

The nontransformed HC11 mouse mammary epithelial cell line used in these studies was obtained as a generous gift from Dr. Wolfgang Doppler (University of Innsbruck). HC11 cells were cultured in complete growth media (CGM) containing RPMI 1640 medium supplemented with 5% fetal bovine serum (FBS), 10 μg/ml bovine insulin, 10 ng/ml epidermal growth factor, 100 U/ml penicillin, 100 μg/ml streptomycin and 500 ng/ml Fungizone. To induce growth arrest by serum and growth factor withdrawal, HC11 cells were cultured to ~80% confluence in CGM and then witched to media deficient in serum and growth factors (growth arrest medium, GAM, 0.1% FBS). To induce growth arrest by contact inhibition, cells were grown to ~80% confluence and maintained in CGM for up to 72 hrs.

Mouse embryo fibroblasts (MEFs) were prepared by harvesting 13.5 day old littermate embryos derived from mating of C/EBPδ +/- heterozygous mice of the C57BL/6 inbred strain. After removing the internal organs and forelimbs, embryos were minced with sterile scissors and incubated with 1 ml of 0.25% (w/v) Trypsin/EDTA solution at 4°C for 6 hours. After incubation, the fibroblast enriched cell preparations were rinsed and cultured in MEF complete growth media (MCGM) composed of DMEM supplemented with 10% (v/v) FBS, 0.2% (v/v) 0.1 M 2-Mercaptoethanol, 1% (w/v) L-Glutamine and 1% (w/v) Penicillin/Streptomycin. MEFS were cultured in MCGM until crisis. Post-crises MEF colonies were isolated and cell lines were established and genotyped as C/EBPδ +/+ or -/-. For growth arrest experiments, MEFS were cultured in media composed of DMEM plus 0.5% FBS plus antibiotics. To induce growth arrest by contact inhibition, MEFs were grown to ~80% confluence and maintained in MCGM for up to 72 hrs. All cultured cells tested negative for the presence of mycoplasma using the Lonza MycoAlert Mycoplasma Detection Kit Cat#: LT07-118 and MycoAlert Assay Control set Cat #: LT07-518.

### C/EBPδ siRNA interference

C/EBPδ RNA interference was induced with short interfering RNA (siRNA) directed against the mouse single exon C/EBPδ gene as previously described [43]. C/EBPδ specific complementary 65 mer oligonucleotides with 5'overhangs were annealed and ligated into pSilencer 2.1-U6 neo SiRNA expression vectors (Applied Biosystems/Ambion). "Scrambled" siRNAs were purchased from Dharmacon (Thermo Scientific). Scrambled siRNAs are nontargeting siRNAs based on genome Blast and microarray analysis performed by Dharmacon. Sequence verified siRNA constructs were transfected into HC11 cells using Lipofectamine (Invitrogen). Stable HC11 cell transfectants were selected by culturing in the presence of with 400 μg/ml G418 for ~15 days. Following selection, stable HC11 cells were maintained in 200 μg/ml G418. C/EBPδ expression levels were assayed by Northern and Western blots. HC11 cells with reduced C/EBPδ expression (>80%) were used for further study.

### Northern blot analysis

Total cellular RNA was isolated from cultured cells using RNA-Bee (Tel-Test, Inc.). Cells were rinsed with PBS and 3.5 ml RNA-Bee was used per 100 mm plate. RNA was precipitated with isopropanol, washed with ice cold 75% ethanol and the RNA pellet dissolved in DEPC water. Total RNA (20 μg) was loaded onto a 1.2% agarose gel and electrophoresed for 2 hr at 150 V. RNA was transferred to Duralon UV membrane (Stratagene), UV cross linked, pre-hybridized and probed with [α-^32^P] dCTP random primer labeled C/EBPδ, β-casein and cyclophilin (CP) cDNAs probes. Hybridization was performed overnight at 42°C. CP was used as a loading control. Results were analyzed from densitometric measurements taken by AlphaImager 2000 (Alpha Innotech). Results are representative of 3 independent experiments.

### Western blot analysis

Western blots were performed as previously described [44]. Cells were rinsed with ice cold PBS, scraped from the plastic plate with a rubber policeman, and lysed in protease containing buffer for 30 mins at 4°C. Total cell lysates were isolated by centrifugation and the soluble supernatant was collected and protein levels quantified by BCA microprotein assay kit (Pierce). Protein lysates (40 μg) were resolved by sodium dodecyl sulfate-polyacrylamide gel electrophoresis (SDS-PAGE), transferred to Immobilon-P PVDF membrane (Millipore), and membranes were blocked for 1 hr with PBS containing 10% non-fat dry milk and 0.5% Tween 20. Membranes were incubated with PBST containing 5% non-fat dry milk and primary antibodies against C/EBPδ (1:1000, rabbit, Santa Cruz), cyclin D1 (1:2500, mouse, Cell Signaling), pSTAT3 (Tyr705) (1:2500, rabbit, Cell Signaling), STAT3 (1:3000, rabbit, Cell Signaling) or β-actin (1:2000, rabbit, Cell Signaling). After washing with PBS, membranes were probed with horseradish peroxidase (HRP)-conjugated anti-rabbit or anti-mouse secondary antibody (1:3000, Cell Signaling) for 1 hr. Membranes were developed using ECL (Pierce) and ECL plus chemiluminescence detection reagent (Amersham Pharmacia). Results are representative of 3 independent experiments.

### [^3^H]-thymidine incorporation assay

The [^3^H]-thymidine incorporation assay was performed as previously described [8]. HC11 cells were seeded in 12-well plates at 9 × 10^4 ^cell/ml in 1 ml CGM and grown to confluence. At the designated time points (0 h, 24 h, 48 h and 72 h), 1 μCi/ml [methyl-^3^H]-thymidine cells was added to the culture media for 2 hr. Cell culture dishes were rinsed twice with PBS, precipitated with ice cold 10% trichloroacetic acid (TCA) for 10 min at 4°C, and solubilized in 0.3N NaOH containing 1% SDS. Incorporation of [methyl-^3^H]-thymidine into TCA precipitated DNA was measured by liquid scintillation counting. Results represent the average of 2 independent experiments with 3 wells/time point.

### Bromodeoxyuridine (BrdU) *In Situ *assay

Cells were plated on 2-well chamber slides at ~9 × 10^4^/ml, grown to near-confluence in CGM and maintained in CGM or GAM. The BrdU incorporation assay was performed using BrdU *In Situ *detection kit (BD Pharmingen). Briefly, 5-bromo-2-deoxyuridine was added to cells with a final concentration of 10 μM/L at the indicated time points and incubated for 2 hrs. Cells were fixed with ice cold methanol at -20°C for 20 min and endogenous peroxidase activity was blocked by incubating slides in 0.3% H_2_O_2 _for 10 min. After heat antigen retrieval, slides were immunostained with biotinylated anti-BrdU antibody and detected by streptavidin-HRP and 3,3'-diaminobenzidine tetrahydrochloride (DAB). Cells were counterstained with hematoxylin for 40 seconds (Vector Lab), cleared with xylene and coverslipped.

### Migration "scratch" assay

HC11 cell migration was assessed by the *in vitro *"scratch" assay as previously described [34,43]. Cells were plated on 100 mm tissue culture plates and grown to confluence. An open area or "scratch" was produced in the HC11 cell monolayer using a 200 μl micropipette tip. HC11 Cells were washed with PBS to remove the displaced cells in the open area, and cultured in CGM or GAM for designated times. MEFs were similarly treated and cultured in MCGM. Migration into the open area was assessed by staining parallel dishes with 0.5% crystal violet in 20% methanol for 3 minutes.

### Cell invasion Assay

Cell invasion assays were performed using the Chemicon Cell Invasion Assay Kit (Chemicon Int.). 1 × 10^6 ^cells were suspended in serum-free media and cultured on the inner (top) chamber of the insert; media with serum was placed in the outer (lower) chamber of the insert. Plates were incubated for up to 6 days at 37°C. Invasive cells which had migrated to the lower surface of the membrane were stained with crystal violet and photographed. Results presented are representative of 3 independent experiments with duplicates.

### HC11 cell differentiation Assay

HC11 cell differentiation assay was performed as previously described [17]. HC11 cells were seeded on 100 mm culture plates and grown to confluence. Confluent cells were washed with PBS twice and maintained in epidermal growth factor (EGF) free RPMI 1640 medium supplemented with 2% FBS, 5 μg/ml insulin, 100 U/ml penicillin, 100 μg/ml streptomycin, and 500 ng/ml fungizone for two days. To induce differentiation 5 μg/ml prolactin (Sigma) and 0.1 μM dexamethasone (Sigma) were added to the medium and cells were cultured for an additional four days. HC11 cells not treated with prolactin and dexamethasone were used as control. RNA and protein samples were isolated as described above.

### Clonogenic Assay

The clonogenic assay was performed as previously described [8]. HC11 cells were transfected with 1 μg of the following constructs cloned into the pcDNA3 expression vector: C/EBPδ cDNA full length (FL), C/EBPδ bZIP domain deleted (DB) or C/EBPδ bZIP + DNA binding domain deleted (TA) constructs. Transfections were performed using Lipofectamine Plus (Invitrogen) and following transfections cells were cultured in complete growth media plus 400 μg/mL G418 (Geneticin). Colonies were stained with a 0.5% crystal violet/20% methanol solution.

### Statistical Analysis

Statistical analysis was performed using Graphpad Prism statistical software. Statistical analysis of treatment group means at multiple time points was performed by one-way analysis of variance (ANOVA). Posttest multiple comparisons of groups means was performed using Tukey's test.

## Abbreviations

C/EBPδ: CCAAT/Enhancer Binding Proteinδ; ChIP: Chromatin Immunoprecipitation; MECs: mammary epithelial cells; MEFs: mouse embryo fibroblasts; CREB: cyclic AMP response element binding protein; Rb: Retinoblastoma protein; CDK: Cyclin dependent kinase; SAGE: Serial Analysis of Gene Expression; CML: chronic myelogenous leukaemia; BRCA1: Breast Cancer 1; VHL: Von Hippel Lindau; BrdU: Bromo deoxyUridine; STAT3: Signaling and Activator of Transcription3; ECM: Extracellular matrix

## Competing interests

The authors declare that they have no competing interests.

## Authors' contributions

All authors contributed to the experimental design, data interpretation, and manuscript development. XY, JS and YZ carried out the experiments, initial data analysis and figure design and optimization. JD advised on all experimental design aspects, data interpretation and final manuscript form.
